# In Search of the Reason for the Breathing Effect of MIL53 Metal-Organic Framework: An ab Initio Multiconfigurational Study

**DOI:** 10.3389/fchem.2017.00111

**Published:** 2017-12-05

**Authors:** Oskar Weser, Valera Veryazov

**Affiliations:** ^1^Institute of Physical Chemistry, University of Göttingen, Göttingen, Germany; ^2^Department of Theoretical Chemistry, Lund University, Lund, Sweden

**Keywords:** metal-organic frameworks, MIL53, phase transition, multiconfigurational methods, spin state, potentialhypersurface

## Abstract

Multiconfigurational methods are applied to study electronic properties and structural changes in the highly flexible metal-organic framework MIL53(Cr). Via calculated bending potentials of angles, that change the most during phase transition, it is verified that the high flexibility of this material is not a question about special electronic properties in the coordination chemistry, but about overall linking of the framework. The complex posseses a demanding electronic structure with delocalized spin density, antifferomagnetic coupling and high multi-state character requiring multiconfigurational methods. Calculated properties are in good agreement with known experimental values confirming our chosen methods.

## 1. Introduction

Metal-organic frameworks (MOFs) are microporous crystalline solids built from inorganic metal centers linked by organic ligands. They have a high area per volume, display large structural diversity and tunable chemical interactions. This offers the possibility to create controlled host-guest interactions and opens new fields in gas storage and separation, drug delivery and especially in catalysis (Férey, 2001; Li et al., 2016). Flexible MOFs, also called soft porous crystals (SPCs) (Horike et al., 2009), are a particular subclass with the possibility of large and reversible structural transformations while preserving all bonds. These transformations, also called “breathing,” can be induced by chemical adsorption of guest molecules, temperature changes and mechanical pressure.

**Figure 1:**
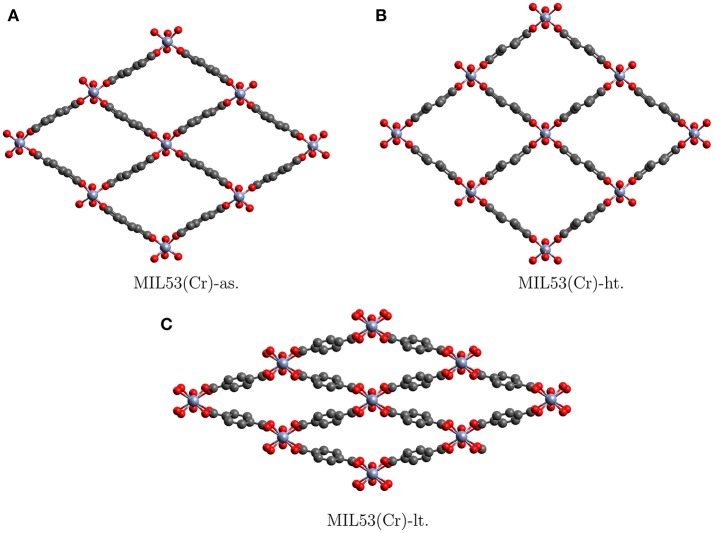
Crystallographic structures of the different allotropes of MIL53(Cr). Hydrogens are not shown (Millange et al., 2002).

One example of SPCs is the MIL53 family. The name was derived from “Matriaux de l'Institut Lavoisier” where they were discovered (Millange et al., 2002). These solids are built from trivalent cations {Cr^III^, Fe^III^, Al^III^} linked by dicarboxylic acids such as *p*-benzenedicarboxylate. They display a large breathing effect of about 50% volume change (Llewellyn et al., 2008), when going from the low temperature form (MIL53-lt) to the high temperature form (MIL53-ht) or the as synthesized (MIL53-as) form. The structures, the large volume change and the similarity between MIL53-ht and MIL53-as can be seen in Figure 1. Apart from temperature the breathing may be initiated and controlled by solvent and gas loading, which makes it very promising for industrial application (Vimont et al., 2007; Hamon et al., 2011; Bousquet et al., 2013; Schneemann et al., 2014). For example MIL53-as still contains solvent molecules from synthesis. Upon heating the solvent evaporates and the pores become empty to give MIL53-ht. After cooling down, water molecules diffuse back into the pores and close them via host-guest interactions to give MIL53-lt. In this context it has to be emphasized that less than 0.05% of all MOFs in the Cambridge Structural Database show substantial breathing in the presence of solvents (Schneemann et al., 2014). So although a lot of the breathing details have to be explained by host-guest interactions, and selectivity toward certain molecules can not be explained without deeper understanding of the intramolecular forces, the flexibility of the framework is in itself a special property (Odoh et al., 2015).

Since discovery the MIL53 family has been subject to several experimental investigations such as diffraction-, adsorption curve and magnetic measurements, IR spectroscopy, and application as drug delivery system (Millange et al., 2002; Serre et al., 2002; Vimont et al., 2007; Horcajada et al., 2008; Liu et al., 2008; Llewellyn et al., 2008). The theoretical treatment involved thermodynamic models for the adsorption (Bousquet et al., 2013), calculation of stress tensors (Ortiz et al., 2012, 2013), host-guest interactions (Coombes et al., 2009; Ma et al., 2012) and several structural calculations accompanying the diffraction experiments.

The stress tensor calculations yielded higly anisotropic Young's moduli which were similar between chemically different, but structurally comparable MOFs: “indicating that their mechanical properties are linked mostly to the nature of their framework, rather than the details of their coordination chemistry” (Ortiz et al., 2013). This leads to the question about the rigidity of the building blocks themselves.

**Figure 2:**
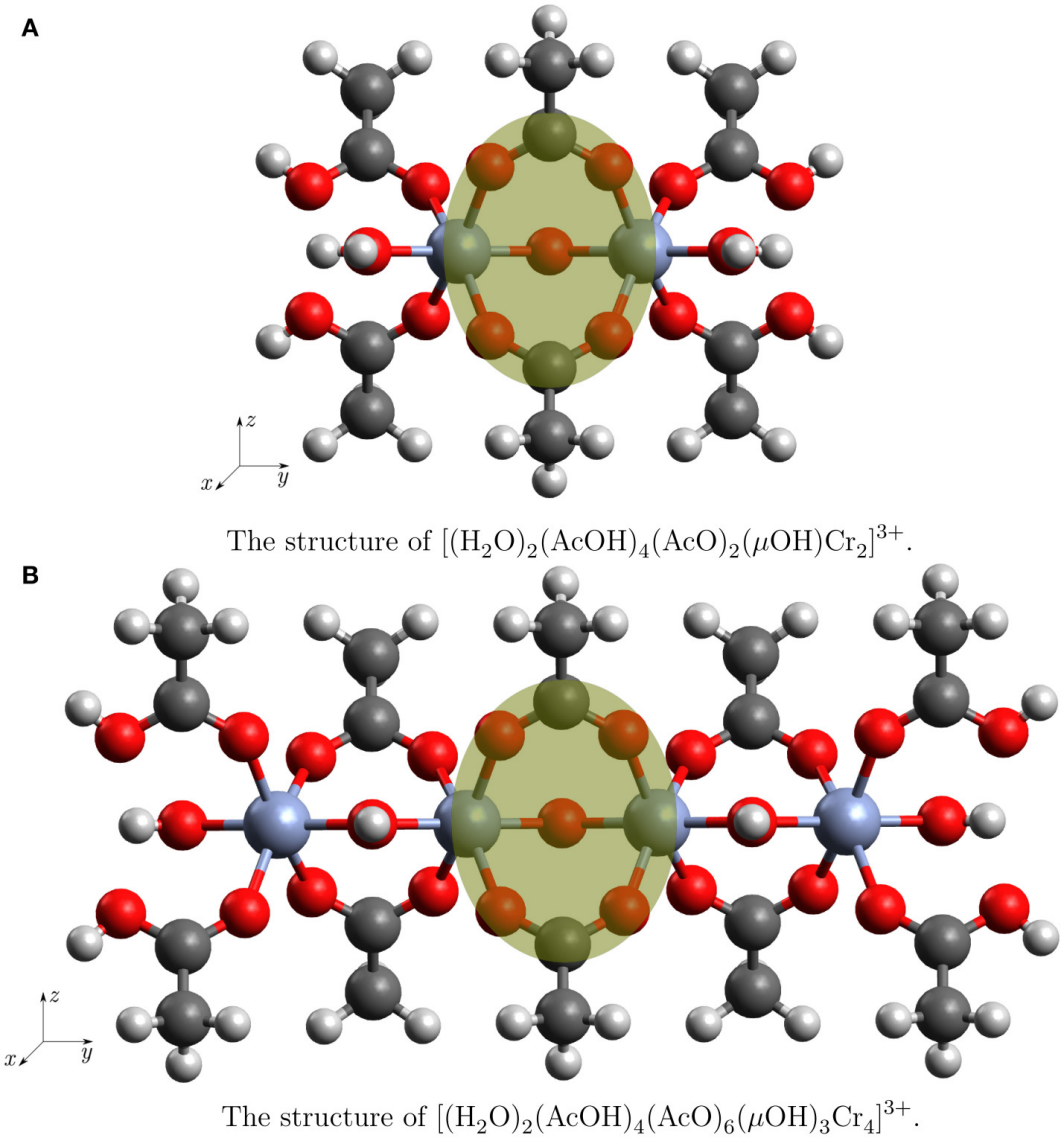
The structures of the clusters used in the truncation test. The atoms in the yellow circle are used to compare the Mulliken charges.

Intuitively one could expect a “floppy” molecule as building block for a crystal with a volume change of 50% while breathing. As it is shown in Figures 2A,B, **4** the Cr^III^-cations are closely connected via two carboxylic groups and a bridging μOH group. The linking of open shell (*d*^3^) transition metal ions via possibly delocalizing ligands could lead to a complicated electronic structure causing an overall shallow potential hypersurface (PHS). To investigate this PHS we were interested about bending potentials for those angles that change the most during transformation from MIL53(Cr)-as to MIL53(Cr)-lt.

Modifying angles of bending potentials of interest broke the lattice's translational symmetry. Hence non-periodic or local methods were applied for calculating the electronic structure. Before doing so it was validated, that truncating the structure does not lead to artifacts.

All previous theoretical calculations on this system were either using periodic density-funcional-theory (DFT) or periodic force fields. This approach describes the periodicity of the electronic structure, but might stumble over the multi-state open shell character of unpaired transition metal *d*-electrons. It is for example known that DFT experiences difficulties with qualitatively describing spin states of complexes with just one transition metal (Pierloot and Vancoillie, 2006, 2008; Lawson Daku et al., 2012; Radoń, 2014). Usually there is at least one functional which reproduces experimental results. But it is nearly impossible to deduce the reliable applicability of a given functional for a given system from intrinsic properties without experimental reference data (Savin, 1996; Pierloot and Vancoillie, 2006, 2008; Lawson Daku et al., 2012; Radoń, 2014; Roos et al., 2016).

In the case of this project however we were interested in bending potentials which require calculations outside the geometric minimum and cannot be measured directly in order to be compared with an experiment. Due to spin and possible multiconfigurational character of MIL53(Cr) a method was needed that is intrinsically suited for such problems, allows “calibration on its own” using systematic basis set expansion and can be applied to large molecular systems. Hence the methods of choice were CASSCF (Complete active space self-consistent field) followed by CASPT2 (Complete active space second-order perturbation theory). They have been shown to give reliable results even in the case of complicated spin structures found in e.g., multi-core transition metal complexes (Azizi et al., 2006; Pierloot and Vancoillie, 2008; Schreiber et al., 2008; Lawson Daku et al., 2012; Roos et al., 2016). In particular it was not possible to apply force field methods to answer the initial question about bending potentials of angles, because they are not a result, but an input parameter of such calculations.

## 2. Methods

### 2.1. Coordinate preparation

Public available cif files of MIL53(Cr) from Millange et al. (2002) were used as starting point for the coordinate preparation. Since the locations of hydrogen were not available from the crystallographic data, the structure of *p*-benzenedicarboxylate was optimized on its own using DFT/B3LYP (Stephens et al., 1994) and the basis set ANO-XS with double zeta quality (Roos et al., 2004, 2005a,b, 2008; Widmark and Veryazov, unpublished). The periodic structure of MIL53 crystal has been approximated as a cluster for the calculations. The covalent C−C bonds, which were broken while constructing the clusters were capped with methyl groups, all other cleaved bonds were capped with protons.

In order to support our procedure of cluster selection, calculations were performed for different system sizes: a smallest one, which contains only two Cr atoms with surroundings and a double size cluster with four Cr atoms. The small cluster with the chemical formula ${\left[{\left({\text{H}}_{2}\text{O}\right)}_{2}{\left(\text{AcOH}\right)}_{4}{\left(\text{AcO}\right)}_{2}\left(\mu \text{OH}\right){\text{Cr}}_{2}\right]}^{3+}$ is shown in Figure 2A. The larger cluster with the chemical formula ${\left[{\left({\text{H}}_{2}\text{O}\right)}_{2}{\left(\text{AcOH}\right)}_{4}{\left(\text{AcO}\right)}_{6}{\left(\mu \text{OH}\right)}_{3}{\text{Cr}}_{4}\right]}^{3+}$ can be seen in Figure 2B. The structures will be referred to as Cr_2_(OR)_9_ and Cr_4_(OR)15 cluster. The Cartesian coordinates are presented in Supplementary Materials. Embedding Cr_4_(OR)_15_ into the electrostatic field of the remaining crystal gave rise to a third cluster which will be referred to as $\left({\text{Cr}}_{4}{\left(\text{OR}\right)}_{15}+\vec{E}\right)$. The field was generated by point charges which had the value of the Mulliken charges obtained from the calculation on Cr_4_(OR)_15_ and were translated by the lattice vectors in positive and negative direction. Structurally, the small cluster is a subset of the larger ones, thus we can use the difference of electronic density in the core parts as indicator for the influence of border effects.

If ρ_1_ and ρ_2_ are the electronic densities of a smaller and bigger cluster, we define: *A* : = $\left\{\text{r}\in {\mathbb{R}}^{3}|0.05{\text{a.u.}}^{-3}\leq \rho_{1}\left(\text{r}\right)\leq 0.50{\text{a.u.}}^{-3}\right\}$ and assume that an increased system size does not affect the valence electronic density of the smaller cluster if the relative change of integrated density difference is negligibly small:

(1) $$\begin{array}{r} \Delta_{rel}\left(\rho \right):=\frac{\underset{A}{\int }|\rho_{1}\left(\text{r}\right)-\rho_{2}\left(\text{r}\right)|\text{d}\text{r}}{\underset{A}{\int }|\rho_{1}\left(\text{r}\right)|\text{d}\text{r}}<\epsilon \end{array}$$

Abstracting Equation (1) we looked into the relative change of ρ_*n*_ under the *L*^1^-norm. We assume that the electronic density ρ_*n*_ depending on the system size *n* converges (under the *L*^1^-norm) to the electronic density of the infinite lattice for *n* → ∞ on any compact set. It directly follows that ρ_*n*_ is a Cauchy-sequence and Equation (1) may be applied physically reasonable to arbitrary system sizes.

Mulliken charges allowed a direct comparison of the chromium ions and its coordination sphere when going from the Cr_2_(OR)_9_ to the Cr_4_(OR)_15_ and (${\text{Cr}}_{4}{\left(\text{OR}\right)}_{15}+\vec{E})$ cluster. The compared atoms are highlighted in Figures 2A,B.

**Figure 3:**
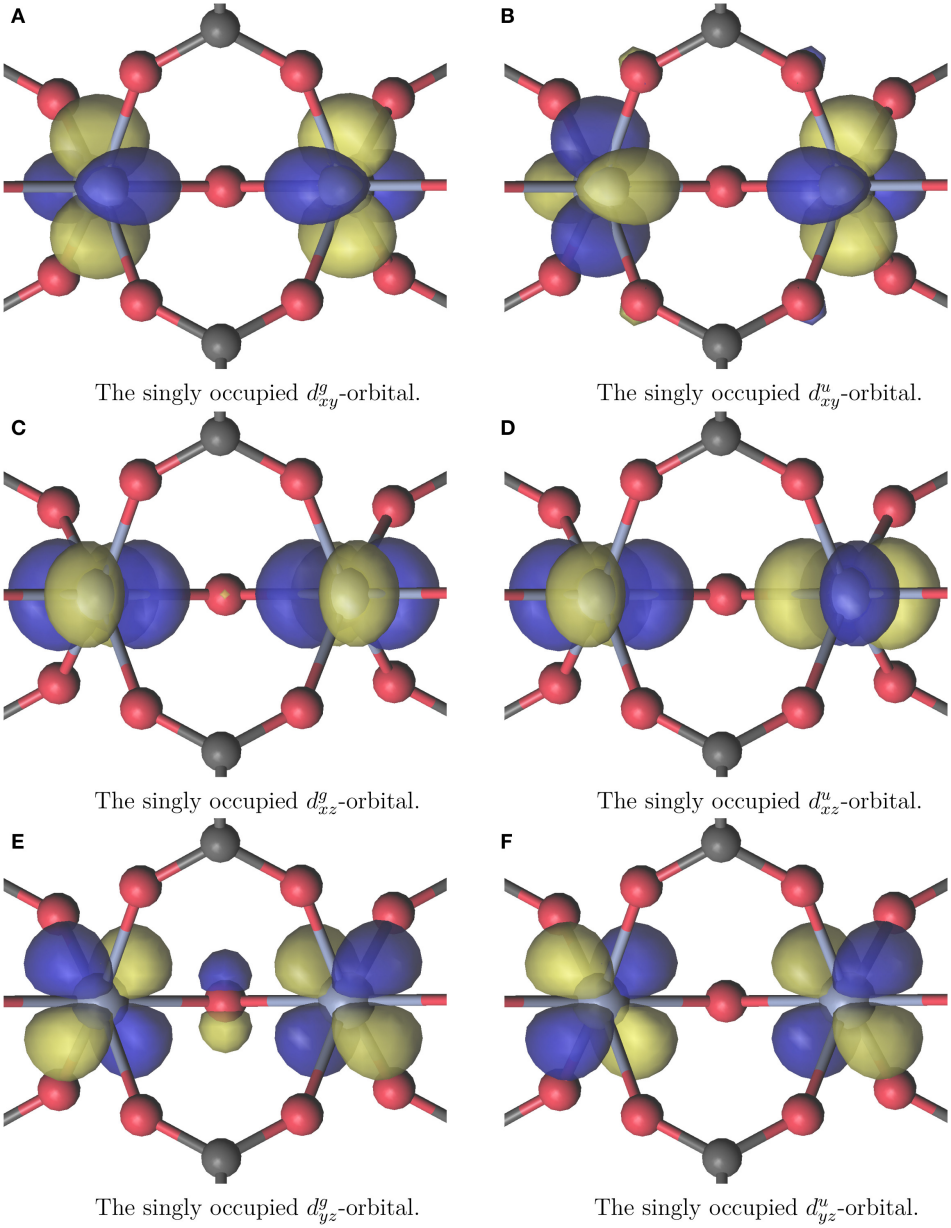
The (6, 6) active space for the CASSCF+CASPT2 calculation on the ${\left[{\left({\text{H}}_{2}\text{O}\right)}_{2}{\left(\text{AcOH}\right)}_{4}{\left(\text{AcO}\right)}_{2}\left(\mu \text{OH}\right){\text{Cr}}_{2}\right]}^{3+}$ cluster displayed in Figure 2A. The *g* and *u* superscripts denote gerade and ungerade linearcombinations. The coordinate systems local to the chromium atoms for denoting the subscripts can be arbitrarily placed.

All calculations were carried out using MOLCAS 8.0 (Karlström et al., 2003; Veryazov et al., 2004; Aquilante et al., 2016). The calculations were made with CASSCF choosing an (3*n*_*Cr*_, 3*n*_*Cr*_) active space where *n*_*Cr*_ is the number of Cr^III^ atoms, thus the triply occupied *T*_*g*_-orbitals of Cr^III^ were included into the active space. The active space of the Cr_2_(OR)_9_ cluster is displayed in Figure 3. It demonstrates the a priori expected single occupation of the *T*_*g*_ orbitals in the octahedral environment of *Cr*. The active spaces of all other calculations look qualitatively similar. Calculations were performed for the anti- and ferromagnetic case. This means the spin states were chosen to be either singlet for both clusters, or septet and 13-let for Cr_2_(OR)_9_ and Cr_4_(OR)_15_ respectively. Relativistic effects were included with the Douglas-Kroll-Hess method (Hess, 1986; Reiher and Wolf, 2004; Peng and Hirao, 2009). The used basis set was ANO-RCC-VDZP on chromium and ANO-XS-VDZ on all other atoms (Roos et al., 2004, 2005a,b, 2008; Widmark and Veryazov, unpublished). All calculations in this work were performed without enforcing symmetry for the wave function, even if the structure belonged to a symmetry group higher than *C*_1_. Artificially enforced symmetry could disguise the favored spin state.

The application of Equation (1) on the calculation's results yields in both coupling cases the same relative change of integrated electronic density Δ_*rel*_(ρ). The values are 3.0% when going from the Cr_2_(OR)_9_ to the Cr_4_(OR)_15_ cluster and 1.4% when going from the Cr_4_(OR)_15_ to the $\left({\text{Cr}}_{4}{\left(\text{OR}\right)}_{15}+\vec{E}\right)$ cluster. The Mulliken charges and their respective changes are summarized in Table 1. Since the clusters have *C*_2υ_ symmetry, some atoms are symmetry equivalent. The average absolute deviation of the Mulliken charges ∅(|Δ_*i*_|) is the same in both coupling cases. The values of ∅(|Δ_*i*_|) are 0.0141 when going from the Cr_2_(OR)_9_ to the Cr_4_(OR)_15_ cluster and 0.0131 when going from the Cr_4_(OR)_15_ to the $\left({\text{Cr}}_{4}{\left(\text{OR}\right)}_{15}+\vec{E}\right)$ cluster. Concluding it may be stated that both indicators: relative change of integrated electronic density and Mulliken charges, show that the applied truncation is valid for this molecule. The detailed information is given in Supplementary Materials. The results show especially that the size dependency is the same for both spin states.

**Table 1:** Mulliken charges for the highlighted atoms in the Cr_2_(OR)_9_ and Cr_4_(OR)_15_ cluster (Figures 2A,B) in the case of antiferromagnetic coupling.

	***n***	**Cr_2_(OR)_9_**	**Δ_1_**	**Cr_4_(OR)_15_**	**Δ_2_**	**$\text{C}{\text{r}}_{4}{\left(\text{OR}\right)}_{15}+\vec{E}$**
Cr	2	1.7370	−0.0180	1.7190	0.0033	1.7223
μO	1	−0.9308	−0.0027	−0.9335	−0.0006	−0.9329
C	2	0.8156	−0.0192	0.7964	−0.0306	0.7658
O	4	−0.7581	0.0125	−0.7456	0.0126	−0.7330
∅(|Δ_*i*_|)			0.0141		0.0132	

*The number of symmetry equivalent atoms is given with n. Δ_1_ is the change from Cr_2_(OR)_9_ to Cr_4_(OR)_15_ and Δ_2_ is the change from Cr_4_(OR)_15_ to ($\text{C}{\text{r}}_{4}{\left(\text{OR}\right)}_{15}+\vec{E}$). The average absolute deviation ∅(|Δ_i_|) is calculated under consideration of n. The results are very similar under ferromagnetic coupling*.

### 2.2. Calculation of potentials

**Figure 4:**
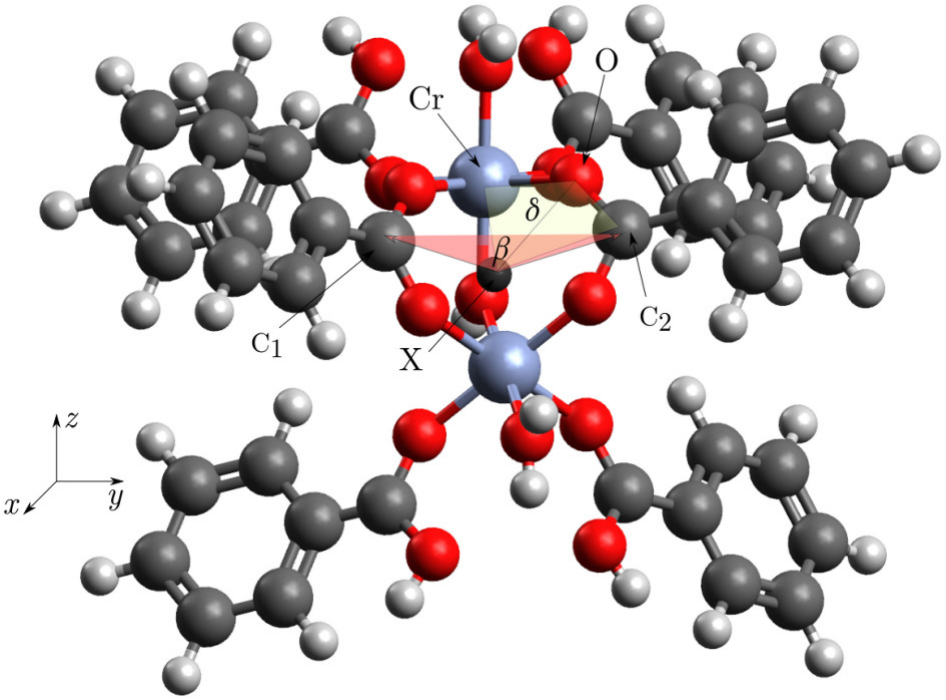
Structure of the cluster for which the potentials were calculated. The labeled atoms define an angle or dihedral which was varied. We defined β: = ∠(C_1_, X, C_2_) and δ: = ∠_dihedral_(*Cr*, O, C_2_, X). The dummy atom X lies between the two Cr-atoms. The bridging OH-group is easier to see in the Figures 2A,B.

Three potential functions were calculated using the structure seen in Figure 4. One bending potential was defined by the angle β : = ∠ (C_1_, X, C_2_), the other one was defined by the dihedral angle δ : = ∠_dihedral_(*Cr*, O, C_2_, X). The dummy atom X was put between the two Cr-atoms. To further validate that the applied truncation gives correct energies away from the potential minimum, the first fundamental of the μOH-stretching mode was calculated and compared with the IR-spectrum by Vimont et al. (2007). A priori it was not clear if the normal mode involving the O−H-distance could be treated as localized in this bond. Since chromium weighs a lot more, than every other atom in this complex it can be safely assumed, that the normal mode is localized into the Cr_2_−O−H entity. Replacing R−Cr_2_ with a virtual particle Y having the mass of the whole remaining complex, gave a three body coupled anharmonic oscillator problem for Y−O−H with two possible stretching modes. This could be treated with vibrational perturbation theory of second order (VPT2) using a quartic force field as described in the work of Stein et al. (2015). The resulting displacements for the two stretching modes showed, that they are localized into the X−O and O−H bond. This meant that the vibrational calculation could be reduced to a one dimensional problem depending only on the O−H-distance, allowing the application of a Morse potential with a closed analytical solution for the vibrational energy.

The electronic structure calculation was carried out with CASSCF and CASPT2 using an (6, 6) active space (Vancoillie et al., 2013). Calculations were performed for the ferro- and antiferromagnetic case. They correspond to a septet and singlet spin state in the truncated system. Relativistic effects were included with the Douglas-Kroll-Hess method (Hess, 1986; Reiher and Wolf, 2004; Peng and Hirao, 2009). All calculations were done with two basis sets: The first one had ANO-RCC-VDZ on chromium and ANO-XS-VDZP on all other atoms and will be referred to as $B_{1}$ (Roos et al., 2004, 2005a,b, 2008; Widmark and Veryazov, unpublished). The other one had ANO-RCC-VTZP on chromium and ANO-XS-VDZP on all other atoms and will be referred to as $B_{2}$ (Roos et al., 2004, 2005a,b, 2008; Widmark and Veryazov, unpublished).

## 3. Results and discussion

**Figure 5:**
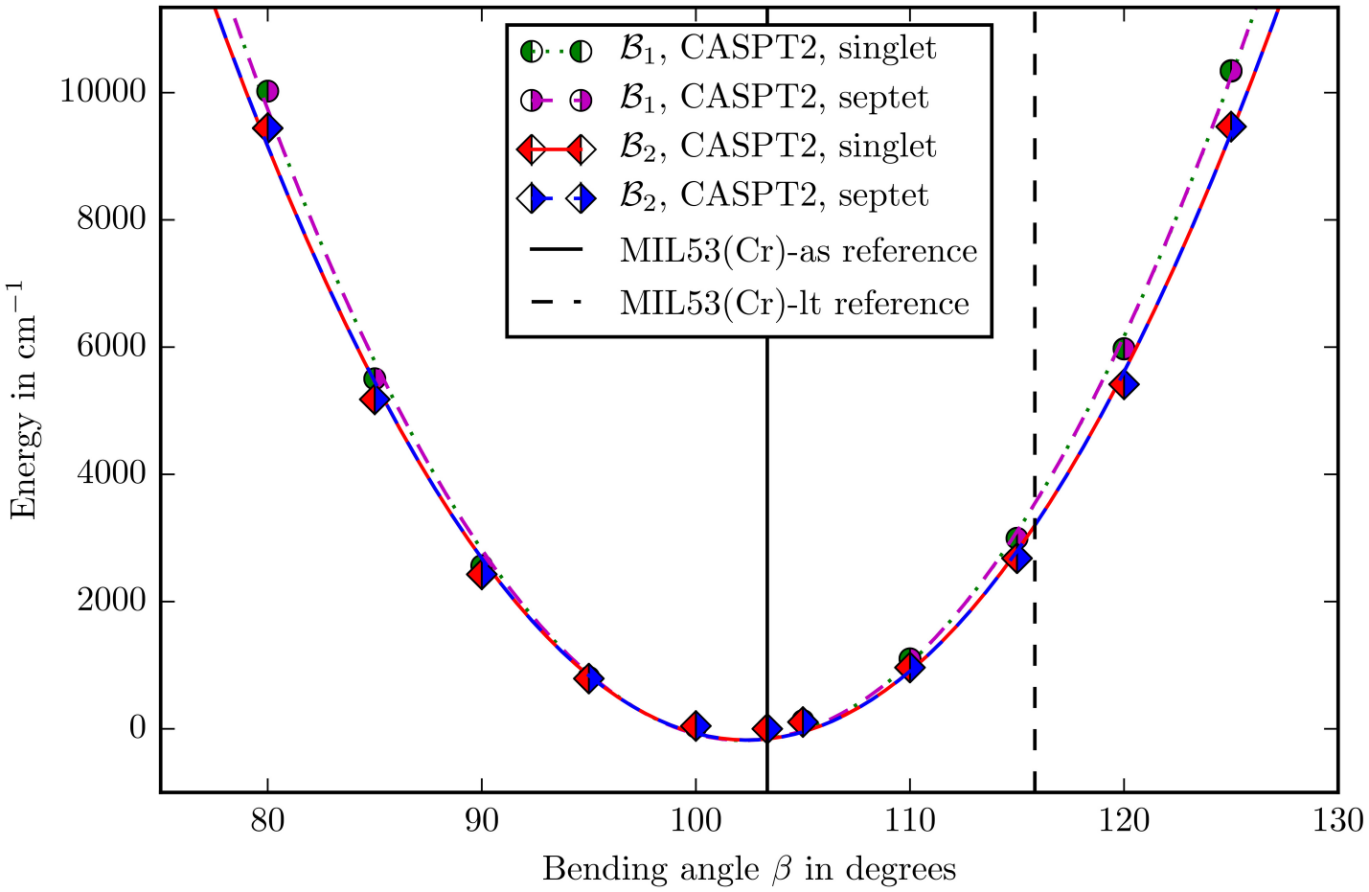
The bending potential for the angle β as defined in Figure 4. The zero point is defined by the energy calculated with the corresponding method and basis set in the reference structure. The points were fitted with a second degree polynomial. The experimental reference values of the angle for the different allotropes are marked with vertical lines (Millange et al., 2002).

**Figure 6:**
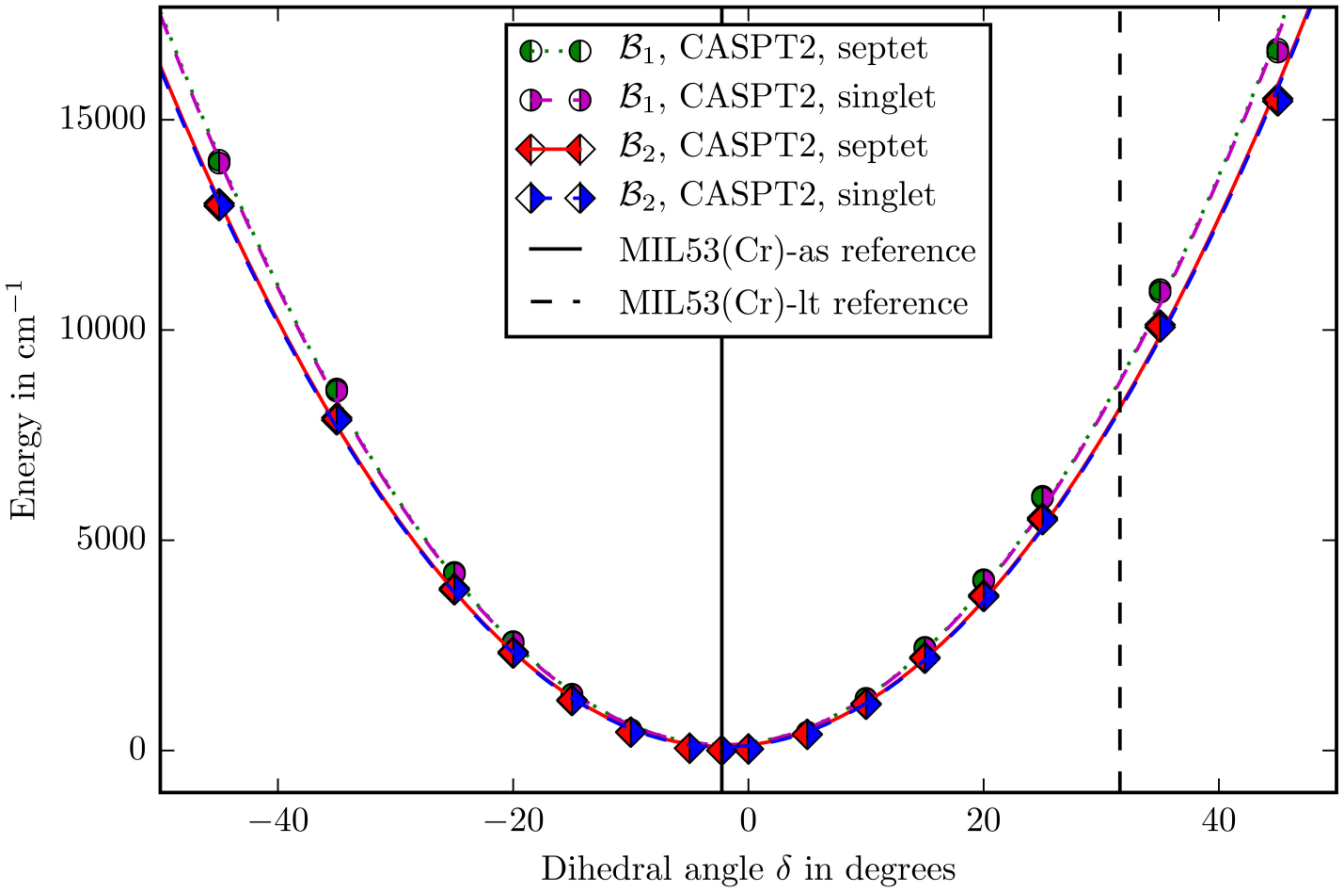
The bending potential for the angle δ as defined in Figure 4. The zero point is defined by the energy calculated with the corresponding method and basis set in the reference structure. The points were fitted with a second degree polynomial. The experimental reference values of the angle for the different allotropes are marked with vertical lines (Millange et al., 2002).

The bending potentials of the angles β and δ are displayed in the Figures 5, 6. For the sake of comparison all potentials were transformed to their own zero point using the energy calculated in the reference structure. All potentials in both figures are well described by a harmonic potential. Hence they were fitted with a second degree polynomial, which yields force constants as single scalar measure for the rigidity of the structure along this coordinate.

The first important question was about the spin state to use. To answer this question, the CASPT2 calculations in the reference structure were compared. The antiferromagnetic state is stabilized by 2.15 kJ/mol using the $B_{1}$ basisset and stabilized by 2.12 kJ/mol using the $B_{2}$ basisset. The energies are per mole Cr-atom. This result is qualitatively consistent with experimentally measured antiferromagnetic behavior (Serre et al., 2002). Unfortunately there is no quantitative experimental data available for the energy difference between the two spin states. From our calculations it may be concluded that the experimental value will lie around 2.0–2.1 kJ/mol. Applying the Mulliken population analysis onto the calculations with the $B_{2}$ basis set yields a spin density of 2.9466 on Cr^III^, 0.0165 on μOH oxygen and 0.0085 on carboxylate oxygen (Compare Figure 4). This result clearly demonstrates the spin delocalization over the ligands.

In the context of spin states, it has to be emphasized that the singlet/antiferromagnetic state is not closed shell. Fourteen Slater-determinants, of which six are in an open shell configuration, contribute with more than 5% to the complete wave function backing the necessity of using multi-configurational methods. The sum of all open shell contributions to the wave function is 50%. It follows that calculations using pure Hartree-Fock or hybrid density functionals are prone to give even qualitatively wrong results. This explains why only functionals with a low fraction of exact Hartree-Fock exchange could be used in the work of Coombes et al. (2009). On the other hand the exchange energy is a necessary part for a reliable description of the electronic structure, so it may be asked if DFT can be used at all to calculate spin states of this complex crystal.

It can be seen in the Figures 5, 6 that, apart from offset, the PHS of the different spin states are very similar. The force constants change by 0.06% in both basis sets when going from singlet to septet. Hence if multi-reference methods are not possible due to computational costs, it may be suitable to assume ferromagnetic coupling for the calculations although it is *not* the electronic groundstate. Most properties of interest are independent from the ofsset of the PHS and the wavefunction of the full spin case does not inherently forbid the use of symmetry and is mostly described by one Slater-determinant.

The second question was about the basis set convergence. The relative change of force constants on the CASPT2 level when changing the basisset $B_{1}$ to $B_{2}$ is 7.2 · 10^−2^ for the angle β and 7.1 · 10^−2^ for δ, which is small enough for making well-founded statements about the rigidity of the molecule in these coordinates.

The final decision about methods and basis sets was to use CASSCF with CASPT2 on the $B_{2}$ basisset (ANO-RCC-VDZP on chromium and ANO-XS-VTZP on every other atom). The spin state was chosen to be singlet. All following discussions imply this setup. A last check was performed for the size of the active space. The *V*(β) potential (Figure 5) was recalculated with an (6, 10) extended active space in the $B_{1}$ basis set. With a change of 0.5% in force constants this influence is negligible and shows that the (6, 6) active space describes the electronic structure well.

Locating the minima in the harmonic fits (Figures 5, 6) gives 102.38° for the β and −2.16° for the δ angle. This reproduces the experimental values of 103.33° and −2.26° quite accurately (Millange et al., 2002).

**Figure 7:**
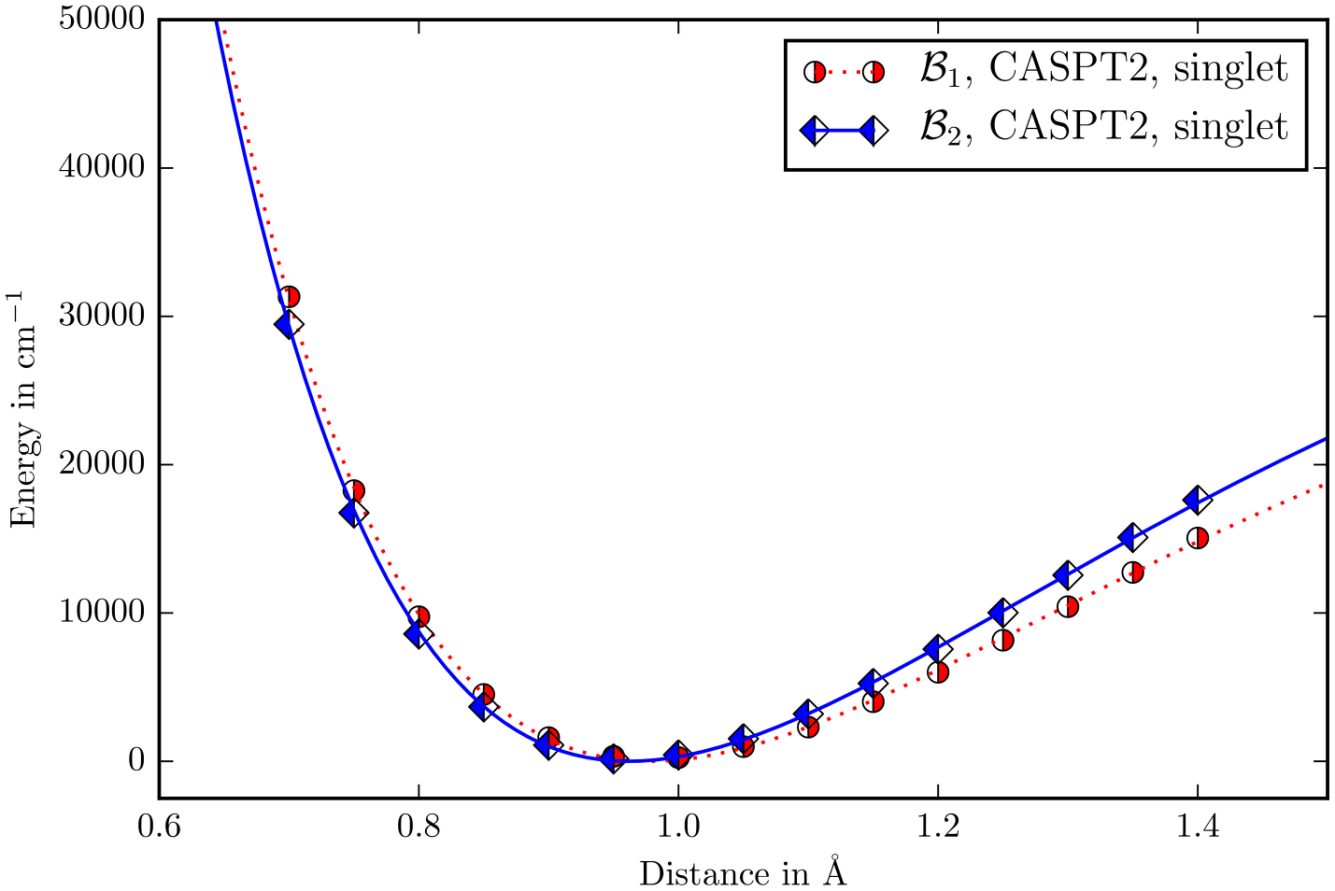
The Morse potential for the O−H-distance in the μOH group. The zero point for every potential is its own minimum. Due to the low scattering cross section of hydrogen in the diffraction experiments, there is no experimental data available.

The perturbative vibrational calculation on the three body problem Y−O−H with a quartic force field showed that the energies of the two stretching modes differ by more than 3, 000cm^−1^. The normal mode with a fundamental of 3, 832cm^−1^ is clearly the measured μOH stretching mode, while the other one with 505cm^−1^ is a skeleton vibration. The displacement in the O−H-distance for the μOH stretching mode is four orders of magnitude higher than the Y−O-distance. It may be concluded that the μOH stretching mode is completely localized into the O−H bond and decoupled from the rest of the molecule allowing to treat it as a one dimensional problem. The calculation of the first fundamental of the μOH stretching mode using a fitted morse potential (Figure 7) gives 3,730 cm^−1^. The relative deviation from the reported experimental value 3,654 cm^−1^ is 2% while it has a halfwidth of ≈100 cm^−1^ (Vimont et al., 2007).

Both the self calibration with basis set expansion and the comparison with experimental values show, that we can trust our results for the bending potentials and answer our question about rigidity of the building blocks.

The angle β changes from 103.33 to 115.83° when going from MIL53(Cr)-as to MIL53(Cr)-lt. Figure 5 shows that if the angle would change on its own, the energy would increase to ≈4,000 cm^−1^ showing counterintuitively that the building block is very rigid in this coordinate.

In the case of δ a similar result is obtained. This coordinate changes from −2.26 to 31.59°. Figure 6 shows that if the angle would change on its own, the energy would increase to ≈9,000 cm^−1^. This shows again that the building block is also very rigid in this coordinate and the “breathing” is not caused by special electronic properties leading to a “floppy” molecule. It occurs, because there are small, coupled movements of the whole framework.

## 4. Conclusion

It was confirmed with preliminary calculations that the periodic structure of MIL53(Cr) may be represented by limited size clusters. That opens the possibility to apply precise ab initio methods developed in quantum chemistry for description of structural changes and corresponding electronic properties in such materials.

Since the complex has a very demanding electronic structure with delocalized spin density, antiferomagnetic coupling and high multi-state character it was necessary to apply multiconfigurational methods like CASSCF followed by second order perturbation theory. To confirm the choice of methods and coordinates, experimental accessible properties were calculated. The located minima of the bending potentials deviate 1° at maximum from the experimental structures of diffraction experiments. The μOH vibrational stretching mode deviates 76cm^−1^ from the experimental peak which has a halfwidth of ≈100 cm^−1^ (Serre et al., 2002).

The important result of this work is that the individual building blocks are indeed quite rigid. The two angles β and δ (as defined in Figure 4) change by 12 and 34° while the structure is breathing. On the other hand the bending potentials expose, that without simultaneous small changes in the whole framework, the energy would increase to ≈4,000 cm^−1^ and ≈9,000 cm^−1^. Consequently it may be concluded that there is no special electronic structure in the coordination environment of Cr^III^ leading to “floppy” molecules. This result further confirms that the interesting mechanical properties of MIL53 are determined by the overall framework. The only possibility for this crystal to breathe is a simultaneous and small change of several angles.

The state-of-the-art multiconfigurational methods and modern software/hardware are capable to study such extended systems, providing high precision for the electronic structure calculations.

## Author contributions

OW performed calculations and wrote the text. VV planned the research and supervised the project.

### Conflict of interest statement

The authors declare that the research was conducted in the absence of any commercial or financial relationships that could be construed as a potential conflict of interest.
