# Supplementary material for: In Search of the Reason for the Breathing Effect of MIL53 Metal-Organic Framework: An ab Initio Multiconfigurational Study
Source: Front Chem. 2017 Dec 5;5:111. doi: 10.3389/fchem.2017.00111 (PMC5723392; doi:10.3389/fchem.2017.00111)
Supplement: Supplementary file 1 [file DataSheet1.PDF]

Supplementary Material:
The breathing effect of MIL53 metal-organic framework

Oskar Weser and Valera Veryazov
*Correspondence:
Oskar Weser, Tammanstr. 6, 37077 Goettingen, Germany
oskar.weser@gmail.com

1 SUPPLEMENTARY DATA

The following files are provided in order to reproduce the results of this paper.

1.1 Files for the $Cr_2(OH)_9$ and $Cr_4(OH)_{15}$ cluster

- `truncation_proof/MIL53as_Cr2(OH)9_cluster.xyz`
  Structure of the $Cr_2(OH)_9$ cluster.
- `truncation_proof/MIL53as_Cr2(OH)9_cluster.png`
  Structure of the $Cr_2(OH)_9$ cluster.
- `truncation_proof/MIL53as_Cr2(OH)9_cluster.InpOrb`
  Starting orbitals for the $Cr_2(OH)_9$ cluster with a $(6, 6)$ active space.
- `truncation_proof/MIL53as_Cr4(OH)15_cluster.xyz`
  Structure of the $Cr_4(OH)_{15}$ cluster.
- `truncation_proof/MIL53as_Cr4(OH)15_cluster.png`
  Structure of the $Cr_4(OH)_{15}$ cluster.
- `truncation_proof/MIL53as_Cr4(OH)15_cluster.InpOrb`
  Starting orbitals for the $Cr_4(OH)_{15}$ cluster with a $(12, 12)$ active space.

1.2 Files for the calculation of the potentials

- `bending_potentials/MIL53as_eq_structure.xyz`
  Reference structure of the cluster used for calculating the potentials.
- `bending_potentials/MIL53as_eq_structure.png`
  Reference structure of the cluster used for calculating the potentials.
- `bending_potentials/MIL53as_beta.molden`
  Movement along the β angle.
- `bending_potentials/MIL53as_delta.molden`
  Movement along the δ angle.
- `bending_potentials/MIL53as_OH.molden`
  Movement of the μOH stretching mode.
- `bending_potentials/B1_basisset_potentials.InpOrb`
  Starting orbitals for the $\mathcal{B}_1$ basis set with a $(6, 6)$ active space.

- `bending_potentials/B2_basisset_potentials.InpOrb`
  Starting orbitals for the $\mathcal{B}_2$ basis set with a $(6, 6)$ active space.
