# Supplementary figures and images for: In Search of the Reason for the Breathing Effect of MIL53 Metal-Organic Framework: An ab Initio Multiconfigurational Study
Source: Front Chem. 2017 Dec 5;5:111. doi: 10.3389/fchem.2017.00111 (PMC5723392; doi:10.3389/fchem.2017.00111)

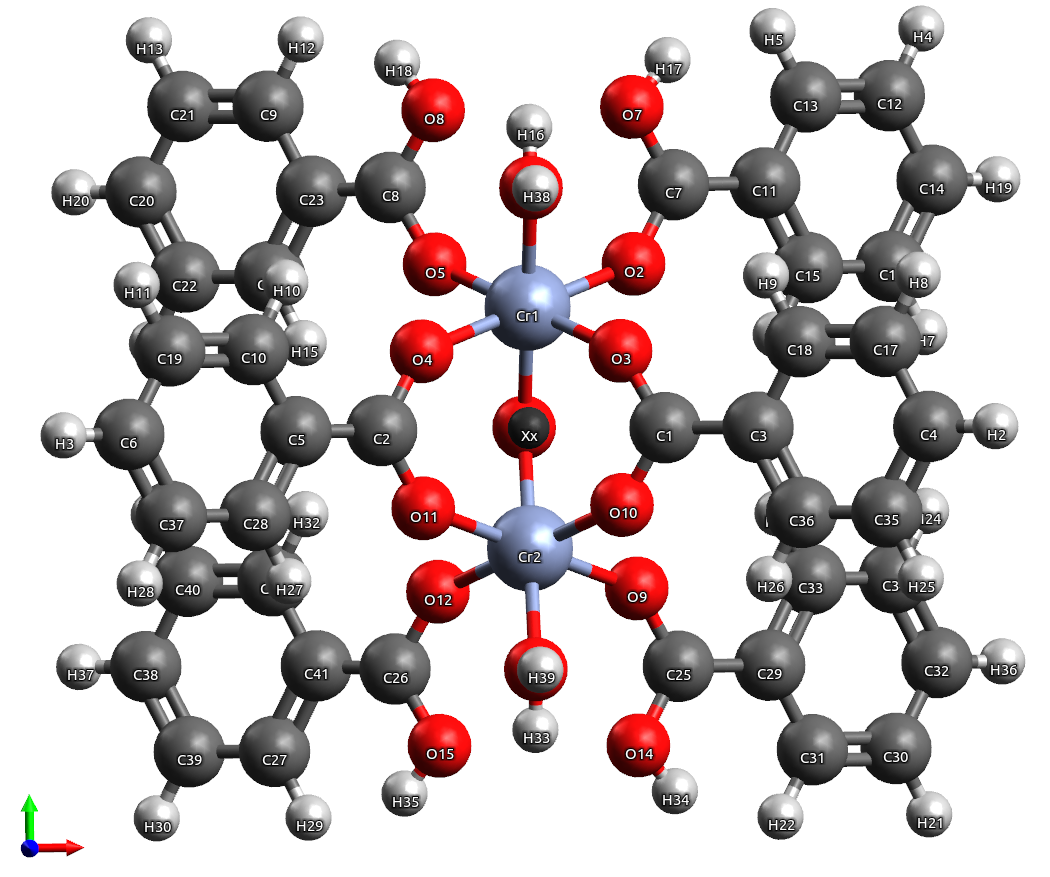

Supplement: Supplementary file 2 [file DataSheet2.ZIP › Attached_files/bending_potentials/MIL53as_eq_structure.png]

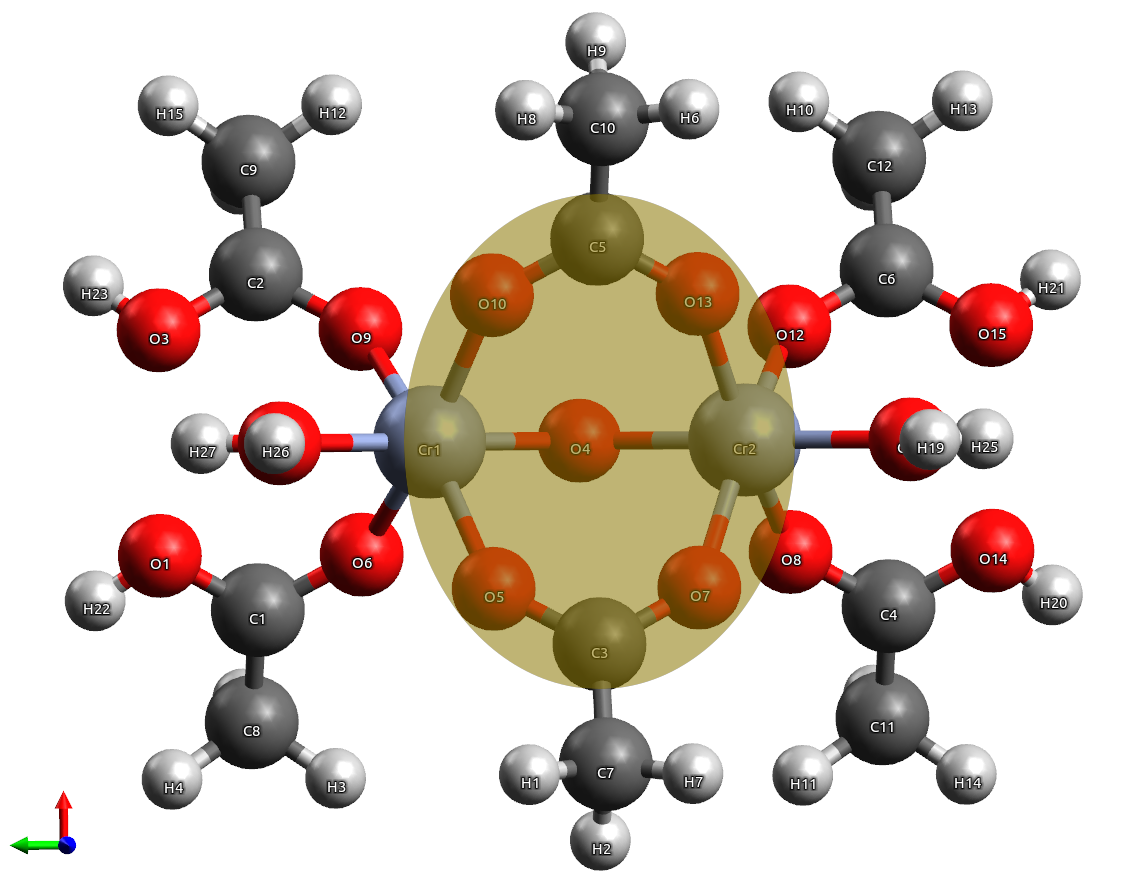

Supplement: Supplementary file 2 [file DataSheet2.ZIP › Attached_files/truncation_proof/MIL53as_Cr2(OH)9_cluster.png]

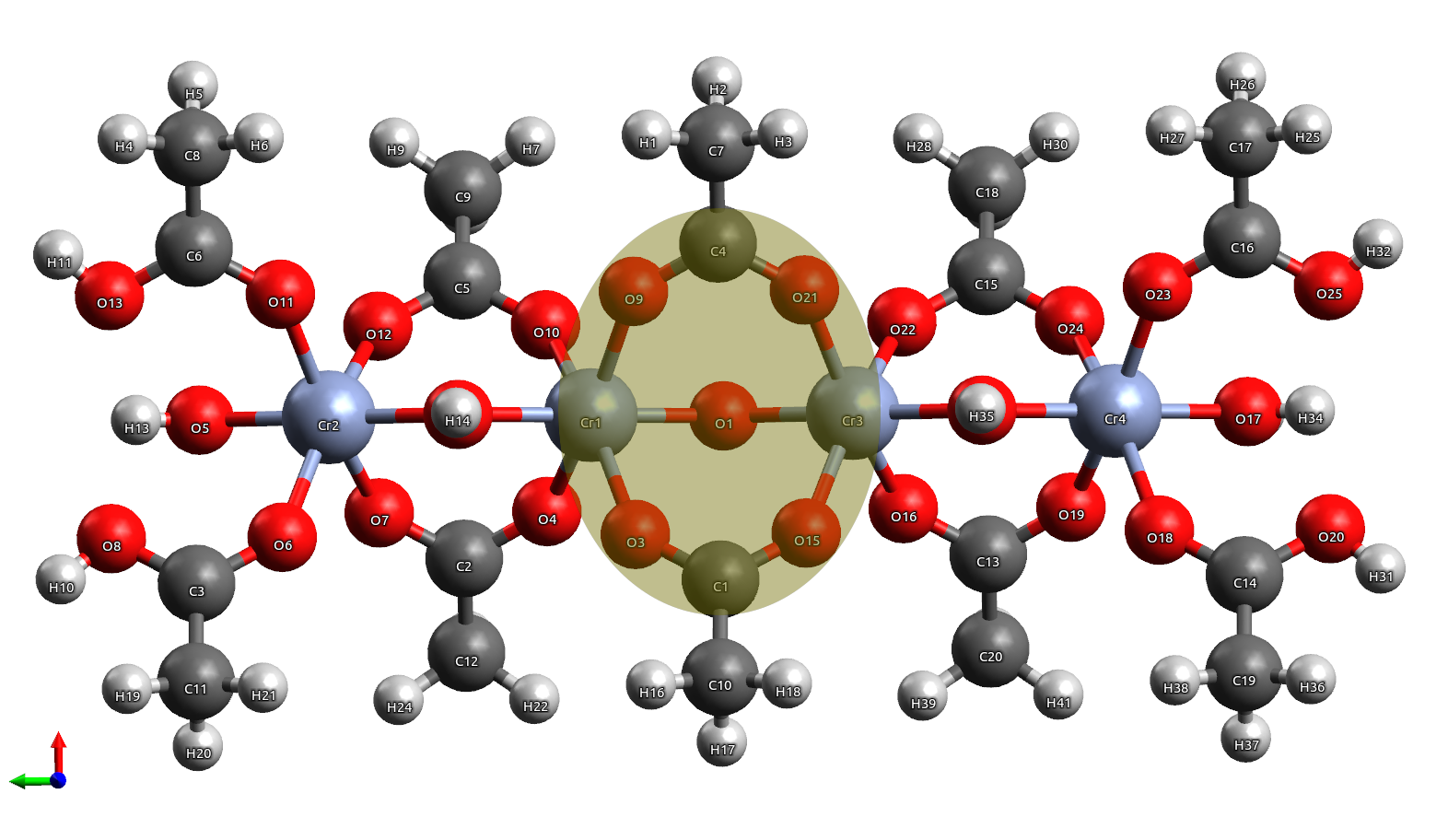

Supplement: Supplementary file 2 [file DataSheet2.ZIP › Attached_files/truncation_proof/MIL53as_Cr4(OH)15_cluster.png]
